# Editorial: Psychophysiological Contributions to Traffic Safety

**DOI:** 10.3389/fnhum.2019.00410

**Published:** 2019-11-19

**Authors:** Guido P. H. Band, Gianluca Borghini, Karel Brookhuis, Bruce Mehler

**Affiliations:** ^1^Leiden Institute for Brain and Cognition, Leiden University Institute of Psychology, Leiden, Netherlands; ^2^Department of Molecular Medicine, Sapienza University of Rome, Rome, Italy; ^3^Neuroelectrical Imaging and BCI Lab, Fondazione Santa Lucia (IRCCS), Rome, Italy; ^4^Faculty of Behavioural and Social Sciences, Groningen University, Groningen, Netherlands; ^5^Massachusetts Institute of Technology, Center for Transportation and Logistics, Cambridge, MA, United States

**Keywords:** attention, traffic performance, human factors, automation, psychophysiology, fatigue, workload

Research shows the dominant contribution of human factors to incidents and accidents in air (Wiegmann and Shappell, 2001), road (Petridou and Moustaki, 2000), rail (Baysari et al., 2009), and maritime traffic participation (Hetherington et al., 2006), as well as in (air) traffic control (Isaac and Ruitenberg, 2016). Operator errors are in majority associated with a non-optimal mental state, such as fatigue, drowsiness, stress, elevated mental workload, distraction from the main task, limited vigilance, and failing situation awareness (Borghini et al., 2014). In turn, most of these functional limitations can be expressed as an aberration of arousal (Collet and Musicant; Lohani et al.) and difficulties maintaining relevant information in working memory (Wu et al., 2017). In an attempt to further reduce traffic casualties, there is an increasing interest in the potential of monitoring the mental state of both professionals and non-professional users. The current Research Topic deals with the question about how mental states can be optimally tracked in simulated as well as naturalistic contexts; now and when technology progresses further toward autonomous driving.

Assessment of fitness to drive by psychometric tools (e.g., self-report such as NASA-TLX; Hart and Staveland, 1988) has serious limitations. Construct validity, sensitivity, and reliability are limited because questionnaires rely on introspection and require a subjective judgment. More importantly, these techniques are not capable of capturing real-time changes, as they are typically not administered during action. Limited gain in traffic safety can be expected from identifying risk only after the fact.

In contrast, dynamic measures have great added value in monitoring the operator's tendencies in real-time during simulated or naturalistic traffic participation. Parameters like steering variability and route compliance (e.g., Getzmann et al.) are directly relevant for operation safety. Similarly, subtle bodily motions can provide clues about the operator's behavioral and muscular tendencies as related to safety. Beggiato et al. showed an increase in backward pressure on the driver's seat when autonomous navigation led to the proximity of a truck. Ihme et al. classified video recordings of facial expressions and were able to identify muscular indicators of frustration, a predictor of less responsible driving. Previous studies have shown the value of tracking head tilt and yawning as indices of drowsiness or fatigue (Reyes-Muñoz et al., 2016).

In contrast with yawning or tightening muscles, which can be perceived as byproducts of mental state, ocular behavior is a functional characteristic that may predict performance. Eye movements reflect overt attention and as such are an index of task-relevant behavior, as defined by areas with and without relevant information. Van de Merwe et al. (2012) demonstrated the value of eye movement parameters such as fixation time, focus and entropy to index situation awareness in simulated flying. Although eye trackers record more than only gaze, other parameters are currently of limited value in naturalistic settings. In well-controlled laboratory or simulator settings, pupillometry has merit in detecting fatigue and workload (Wiegand et al., 2008). However, the pupil's strong response to variable lighting makes it virtually impossible to recognize subtle pupil dilation associated with arousal levels under naturalistic conditions. Eye blink duration, however, has successfully been linked to workload and fatigue (Benedetto et al., 2011).

In comparison with behavioral tendencies, psychophysiological and neuroimaging indicators tap even more directly into mental states during traffic participation (Van Erp et al., 2015; Borghini et al., 2017b). They have the potential to detect adverse changes before a change in the user functional state is visible in behavior. Near infrared spectroscopy (NIRS) is successful at recognizing frustration (Ihme et al.) and elevated workload (Le et al.; Scheunemann et al.) with accuracy of classification ranging from 78 to 90%. Electroencephalographic activity (EEG) has traditionally been used to distinguish spectral contributions associated with higher cognitive activity (Borghini et al., 2017a; Di Flumeri et al., 2018) vs. sleep-like activity (Simon et al., 2011; Fonseca et al., 2018). EEG is superior to blood-oxygenation based recordings in its temporal resolution, which allows for the identification of transient stimulus-induced changes using event-related potentials (Brookhuis and de Waard, 2010; Rupp et al., 2019) or time-frequency analyses (Gurudath and Riley, 2014).

Mental states are not only reflected in brain activity, but also in the activity and balance of the autonomic nervous system. In particular, arousal, vigilance (Schmidt et al., 2009), and fatigue (Wang et al., 2018) can be tracked with cardiovascular recording techniques (see Lohani et al. for a review). In addition, electrodermal activity can index elevated mental workload (Mehler et al., 2012) and stress (Boucsein, 2012). As more psychophysiological signals are monitored, the reliability of estimating the user's mental state can only improve (Sahayadhas et al., 2012).

Neuroscientific methods have long suffered from practical limitations, such as non-portability, intolerance to motion, invasive or intruding sensors, and computational demands that prohibited real-time use. With the advance of technology, however, more and more of these neuroscientific approaches become accessible for real-time applications, and occasionally also for improving real-world traffic safety, as in driver assistance systems. Unfortunately, not all affordable sensors are suited to monitor performance potential. Cisler et al. showed that high-tech EEG recordings of midline alpha band could model speed of responding to faulty behavior of an autonomous car, but that low-tech indicators of eye gaze and heart-rate variability lacked predictive power.

The collection of papers in this Research Topic illustrates current topics in transportation research. Technology is moving forward. This introduces the challenge to maintain safety in the context of the increasingly popular, but yet imperfect operator assistance and automated driving systems. At the same time, new technology is rapidly providing new hardware, data processing algorithms and artificial intelligence that may make it more feasible and acceptable to track the operator's mental state and actively support, as needed, situation awareness. We know from the relative successes in aviation that it is possible to keep pilots aware and capable of taking over control despite extensive use of the autopilot mode. An important challenge is now to reach a similar level of capability in non-professionals, even in adverse conditions. Psychophysiological techniques can play a critical role in achieving this goal.

## Author Contributions

All authors listed have made a substantial, direct and intellectual contribution to the work, and approved it for publication.

### Conflict of Interest

The authors declare that the research was conducted in the absence of any commercial or financial relationships that could be construed as a potential conflict of interest.
